# A Review on Using Crumb Rubber in Reinforcement of Asphalt Pavement

**DOI:** 10.1155/2014/214612

**Published:** 2014-01-30

**Authors:** Nuha Salim Mashaan, Asim Hassan Ali, Mohamed Rehan Karim, Mahrez Abdelaziz

**Affiliations:** Center for Transportation Research, Faculty of Engineering, University of Malaya, 50603 Kuala Lumpur, Malaysia

## Abstract

An immense problem affecting environmental pollution is the increase of waste tyre vehicles. In an attempt to decrease the magnitude of this issue, crumb rubber modifier (CRM) obtained from waste tyre rubber has gained interest in asphalt reinforcement. The use of crumb rubber in the reinforcement of asphalt is considered as a smart solution for sustainable development by reusing waste materials, and it is believed that crumb rubber modifier (CRM) could be an alternative polymer material in improving hot mix asphalt performance properties. In this paper, a critical review on the use of crumb rubber in reinforcement of asphalt pavement will be presented and discussed. It will also include a review on the effects of CRM on the stiffness, rutting, and fatigue resistance of road pavement construction.

## 1. Introduction 

Roadways are an integral aspect of transportation infrastructure. Road construction engineers must consider the primary user's requirements of safety as well as the economy. To achieve this goal, designers should take into account three fundamental requirements which include environmental factors, traffic flow, and asphalt mixtures materials [1–3]. In asphalt concrete (AC), bitumen as a binder serves two major functions in road pavement, first, to hold the aggregates firmly and second to act as a sealant against water. However, due to some distresses like fatigue failure, the performance and durability of bitumen are highly affected by changes with time in terms of its characteristics which can lead to the cracking of pavement [2]. In general, road pavement distresses are related to asphalt binder (bitumen) and asphalt mixture properties. Rutting and fatigue cracking are among the major distresses that lead to permanent failure of the pavement surface. The dynamic properties and durability of conventional asphalt, however, are deficient in resisting pavement distresses. Hence, the task of current asphalt researchers and engineers is to look for different kinds of polymer modified asphalt such as crumb rubber [3]. The term reinforced pavements refers to the use of one or more reinforcing layers within the pavement structure. Another application of pavement reinforcement is the use of reinforcement elements in asphalt overlays to provide an adequate tensile strength to the asphalt layer and to prevent failures of the pavement such as reflection cracking. Thus the difference between the two applications is that the first application is used as measure to overcome the distress failure which already occurred in the pavement, while the second application is used as measure to prevent the existence of such failure. Modification/reinforcement of asphalt binder is possible during different stages of its usage, either in between binder production and mix processes or before paving mix production [4]. According to Larsen et al. [5] the bitumen modification provides binders with:

- sufficient increase in consistency at the highest temperature in pavements to prevent plastic deformation,
- an increase in flexibility and elasticity of binders at low temperature to avoid crack deformations and loss of chippings,
- an improvement of adhesion to the bitumen into aggregates,
- improved homogeneity, high thermo stability, and aging resistance which helps reduce the hardening and initial aging of the binders during mixing and construction.

Worldwide, there are many additives used as reinforcing material into the asphalt mixes, among these additives used is the CRM [3, 4]. In this paper, asphalt pavement design criteria will be displayed and a considerable review on the use of crumb rubber in asphalt pavement reinforcement will be presented and discussed. It also includes a review on the effects of CRM on the stiffness, rutting, and fatigue resistance of road pavement. In order to understand the asphalt-rubber reinforcement technology, asphalt properties and crumb rubber characteristics will be illustrated.

## 2. Asphalt Pavement Design 

**Figure 1:**
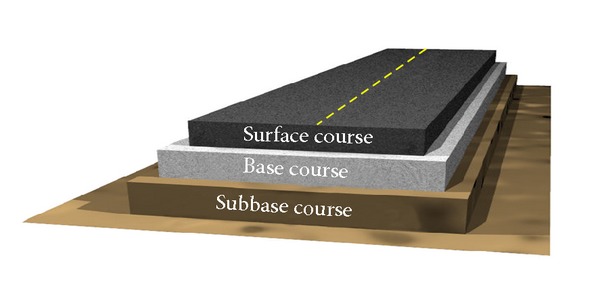
Typical flexible pavement structure.

The design of asphalt mixture involves the selection and proportioning of materials to obtain the desired properties in the finished product. Asphalt concrete (AC) is designed to resist rutting, fatigue, low temperatures cracking and other distresses. The serious distresses associated with asphalt pavements are cracking, which occurs at intermediate and low temperatures, and permanent deformation, which occurs at high temperatures. These distresses reduce the services life of the pavement and elevate the maintenance costs [6]. Asphalt cement binds the aggregate particles together, enhancing the stability of the mixture and providing resistance to deformation under induced tensile, compressive, and shear stresses. The performance of asphalt mixture is a function of asphalt cement, aggregate, and its volumetric properties. In recent years, there has been a rapid increase in using additives in asphalt concrete mixtures to improve its properties. Asphalt road pavements are defined as asphalt layers built bound over a granular base. Due to this, the total pavement structure deflects due to traffic loads, thus these types of pavements are known as flexible pavements. A flexible pavement structure is composed of various layers of materials. Basically, the pavement structure is divided into three layers, namely, bituminous surfacing (surface course), road base (base course), and subbase [6], as shown in Figure 1.

**Figure 2:**
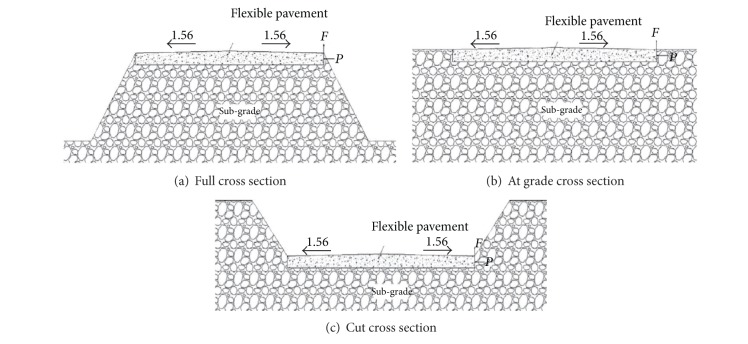
Flexible pavement typical cross section geometries [7].

Flexible pavements could have one of the three typical cross section geometries as shown in Figure 2. At the pavement edge, between the pavement edge and adjacent soil two forces exist which are vertical friction, *F*, and lateral passive pressure, *P*. The friction force (*F*) relies on relative movement, coefficient of friction, and the lateral passive pressure. Lateral passive pressure (*P*) varies depending on soil type and weight of the soil subjected to the pavement. As illustrated in Figure 2(a), the soil wedge is small and the two forces (*F* and *P*) can be ignored. On the other hand, as shown in Figures 2(b) and 2(c), the friction and passive forces may be significant and the pavement edge can move laterally and vertically [7].

**Figure 3:**
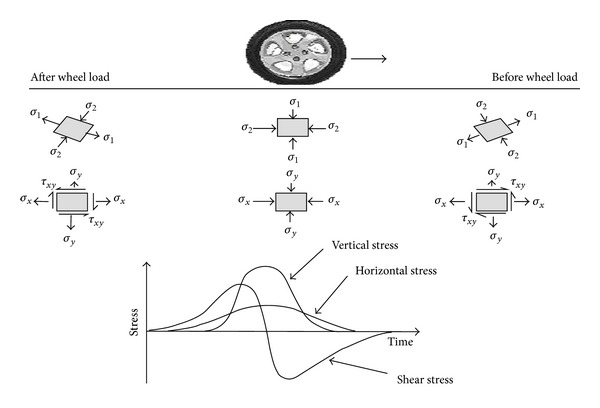
Stress beneath a rolling wheel load [8].

Asphalt concrete (AC) should have high stiffness to be able to resist permanent deformation. On the other hand, the mixtures should have enough tensile stress at the bottom of the asphalt layer to resist fatigue cracking after many load applications.  Figure 3 presents the orientation of principal stresses with respect to position of rolling wheel load [8].

The overall objective for the design of asphalt paving mixes is to determine an economical blend and gradation as well as asphalt binder that will yield a mix having sufficient binder to ensure a durable pavement, sufficient stability, sufficient voids in the total compacted mix to allow for a slight amount of additional compaction under traffic loading without flushing, and sufficient workability to permit efficient placement of the mix without segregation [9].

The increased demand on highway roads might reduce its strength properties and make roads more susceptible to permanent distresses and failure. In general, pavement performance properties are affected by the bitumen binder properties; it is known that the conventional bitumen has a limited range of rheological properties and durability that are not sufficient enough to resist pavement distresses. Therefore, bitumen researchers and engineers are looking for different types of bitumen modifiers. There are many modification processes and additives that are currently used in bitumen modifications such as styrene butadiene styrene (SBS), styrene-butadiene rubber (SBR), ethylene vinyl acetate (EVA), and crumb rubber modifier (CRM). The use of commercial polymers such as SBS and SBR in road and pavement construction will increase the construction cost as they are highly expensive materials. However, with the use of alternative materials such as crumb rubber modifier (CRM), it will definitely be environmentally beneficial, and not only it can improve the bitumen binder properties and durability but it has also a potential to be cost effective [10–12].

## 3. Historical Experiment of Using Crumb Rubber in Pavement 

In 1840s, the earliest experiments had involved incorporating natural rubber into asphalt binder to increase its engineering performance properties. The process of asphalt modification involving natural and synthetic rubber was introduced as early as 1843 [13]. In 1923, natural and synthetic rubber modifications in asphalt were further improved [14, 15]. According to Yildirim [15] the development of asphalt-rubber materials being used as joint sealers, patches, and membranes began in the late 1930s. The first attempt to modify asphalt binders by adding rubber was made in 1898 by Gauedmberg, who patented a process for manufacturing asphalt rubber. France was then given credit for installing the first road with a crumb rubber modified asphalt surfacing material [2].

In 1950, the use of scrap tyre in asphalt was reported [16]. In the early 1960s, Charles Mc Donald working as head Material Engineer for the city of Phoenix, Arizona, found that, after completing the mixing of crumb rubber with the virgin asphalt cement and allowing it to blend for mix duration of 45–60 minutes, there were new material properties produced. There was swelling in the size of the rubber particles at higher temperatures allowing for higher concentrations of liquid asphalt contents in pavement mixes [17]. The application of rubber-modified asphalt pavement started in Alaska in 1979. Placement of seven rubberised pavements totalling 4 lane km using the Plus Ride dry process between 1979 and 1981 was reported. The performance of these sections in relation to mixing, compaction, durability, fatigue, stability and flow and tyre traction and skid resistance were described. Asphalt rubber using the wet process was first applied in Alaska in 1988 [18]. Around 1983 in the Republic of South Africa, asphalt-rubber seals were first introduced. Over 150 000 tons of asphalt were paved over the first 10 years. From the evaluation, it was concluded that the asphalt rubber stress absorbing membrane interlayers (SAMIs) and asphalt performed above expectations. The asphalt rubber overlays out-performed the virgin asphalt, under identical conditions, by a large margin. Asphalt-rubber and SAMIs are especially suited for highly trafficked roads with pavements in structural distress and where overlays will eliminate reworking options in congested traffic situations [19]. Lundy et al. [20] presented three case studies using crumb rubber with both the wet process and dry process at Mt. St. Helens Project, Oregon Dot and Portland Oregon. The results showed that even after a decade of service crumb rubber products have excellent resistance to thermal cracking. Although, asphalt-rubber mixtures can be built successfully, quality control ought to be maintained for good performance. Rubber Pavement Association found that using tyre rubber in open-graded mixture binder could decrease tyre noise by approximately 50%. Also, in spray applications, rubber particles of multiple sizes had better sound absorption [21]. Moreover, another advantage of using asphalt rubber is to increase the life span of the pavement. However, recommendations were made to assess the cost effectiveness of asphalt rubber [22]. The benefits of using crumb rubber modified bitumen are lower susceptibility to varying temperature on a daily basis, more resistance to deformation at higher pavement temperature, proved age resistance properties, higher fatigue life for mixes, and better adhesion between aggregate and binder. Ever since then, the use of crumb rubber has gained interest in pavement modification as it is evident that crumb tyre rubber can improve the bitumen performance properties [23–26].

In Malaysia, the use of rubber as an additive for road pavement construction supposedly started in the 1940s, but there has not been any official record of such practices. The first recorded trial using rubberised bitumen technology was reported in 1988, the wet mix process was used with the mix of rubber additives in the form of latex into bitumen binder [27]. In 1993, another rubberised road trial using waste gloves and natural rubber latex was carried out in Negeri Sembilan [28].

## 4. Interaction Mechanism of Asphalt Rubber Elements

Previous researchers found that when incorporating the rubber powder into asphalt cement the rubber will degrade and its effectiveness is reduced on prolonged storage at elevated temperatures [2]. The improvements effected in the engineering properties of Asphalt Rubber (AR) depend largely on the particle dispersion, the molecular level dissolution, and the physical interaction of rubber with asphalt. Temperature and time of digestion are highly important factors affecting the degree of dispersion for slightly vulcanized and vulcanized natural rubber. For instance, the optimum digestion time for a slightly vulcanized rubber powder is 30 minutes at 180°C and 8 h at 140°C [29]. On the other hand, vulcanized rubber powder requires merely 10 minutes digestion time at 160°C to achieve the same results. The easy dispersion of unvulcanised powder is because of the state of the rubber and fineness of the powder (95 percent passing 0.2 mm sieve). Vulcanised powders are harder to disperse because they are coarser (about 30 percent retained on 0.715 mm sieve and 70 percent retained on 0.2 mm sieve) and also due to vulcanization. According to Jensen and Abdelrahman [30] there are three stages of interaction that have been evaluated with regard to asphalt rubber binder: (i) an early stage that occurs immediately after mixing crumb rubber with bitumen; (ii) an intermediate storage stage, during which the binder is held at elevated temperatures for up to a few hours before mixing with aggregate; (iii) an extended (storage) stage when bitumen-rubber blends are stored for extended periods before mixing with aggregate. Miknis and Michon [31] investigated the application of nuclear magnetic resonance imaging to rubberised bitumen binder. The application of this technology led to investigating the different interaction between crumb rubber and asphalt such as swelling by asphalt molecules, possible dissolution of rubber components in asphalt, and devolatitisation and crosskicking in rubber. The outcome of this study is swelling of rubber particles which may depend on asphalt molecules. According to Shen et al. [32] the factors which affect the digestion process of the asphalt and rubbers blends are rubber content, rubber gradations, binder viscosity, binder source, and blending conditions of time and temperature.

## 5. Key Factors That Influence the Properties of Asphalt Rubber 

### 5.1. Asphalt Properties

**Figure 4:**
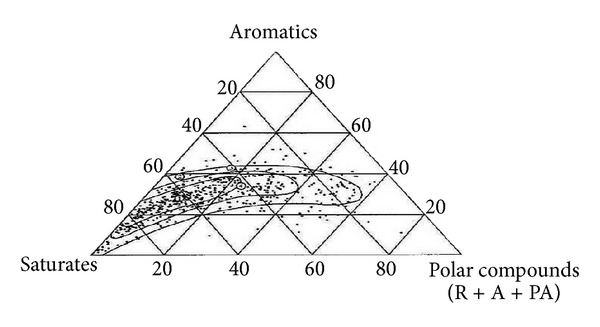
Compositional representation on ternary diagram of 640 different crudes [36].

**Figure 5:**
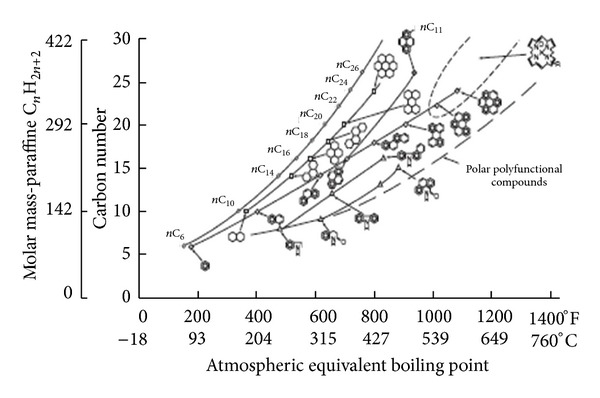
Evolution of molecular weights and structures as a function of the boiling point [38].

Asphalt is a dark black semisolid material, obtained from the atmospheric and vacuum distillation of crude oil during petroleum refining which is then subjected to various other processes [33]. It is considered as a thermoplastic viscoelastic adhesive which is used for road and highway pavement engineering, primarily because of its good cementing power and waterproof properties [34]. The analysis of bitumen indicates that the mix is approximately 8–11% hydrogen, 82–86% carbon, 0–2% oxygen, and 0–6% sulphur by weight with minimal amounts of nitrogen, vanadium, nickel and iron. In addition, it is a complex mixture of a wide variety of molecules: paraffinic, naphthenic, and aromatics including heteroatoms [34]. Most producers use atmospheric or vacuum distillation to refine the asphalt cement. While there is some solvent refining and air blowing utilised, they are clearly of secondary importance [35]. Based on chemical analysis, crude oil may be predominantly paraffinic, naphthenic, or aromatic with of paraffinic and naphthenic combinations being most common. There are approximately 1500 different crudes produced globally. According to the yield and quality of the resultant product, only a few of these, presented in Figure 4 (compositions are in percentage of weight and represent the +210°C fraction), are considered appropriate for the manufacture of bitumen [36, 37]. The most commonly used method and probably the oldest method is the atmospheric vacuum distillation of suitable crudes which produce straight-run residual asphalt. The air blowing process is done to give oxidized or semiblown products, which are inherently upgrades of low-grade asphalt. Crude heavy fractions are defined as molecules containing more than 25 carbon atoms (C25), which increases with the boiling point (Figure 5) as well as the molecular weight, the density, the viscosity, the refractive index (aromaticity) and the polarity (contents of heteroatoms and metals) [38, 39]. These fractions are enriched in highly polar compounds such as resins and asphaltenes. When compared to the crude or lighter fractions, the highly polar compounds are composed of various chemical species of different aromaticity, functional heteroatoms, and metal contents [38, 39].

#### 5.1.1. Asphalt Chemical Components

**Figure 6:**
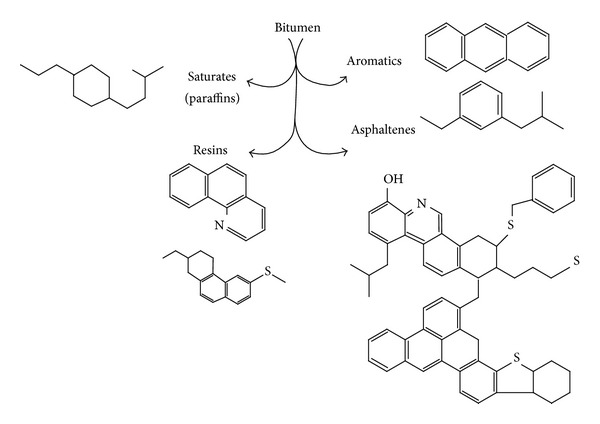
Representative structures of asphalt fractions [43].

The chemical component of asphalt cement can be identified as asphaltenes and maltenes. The maltenes can be further subdivided into three groups of saturated, aromatic, and resins. The polar nature of the resins provides the asphalt its adhesive properties. They also act as dispersing agents for the asphaltenes. Resins provide adhesion properties and ductility for the asphalt materials. The viscous-elastic properties of asphalt and its properties as a paving binder are determined by the differing percentages between asphaltenes and maltenes fraction [40–42].  Figure 6 shows the representative structures of four generic groups (SARA): saturated, aromatic, resins (which are form the maltene fraction), and asphaltenes. This model is based on the colloidal model [43, 44]. The complexity, content of heteroatom, aromatic, and increase of molecular weight are in the order of S < A < R < A (saturates < aromatics < resins < asphaltenes) [45]. A study by Loeber et al. [46] illustrated the rheological properties related to asphalt colloidal behaviour. Also, it possesses a strong temperature dependence on rheological properties organised by the interaction of individual constitution (asphaltenes, resins, aromatics, and saturates). Loeber et al. [46] reported that an increase in one of these constitutions would change the structure and rheological behaviour of asphalt cement. Thus, asphalt with high asphaltenes/resins ratio would lead to a network structure with more rigidity and elasticity (low in phase angle and high in complex shear modulus), unlike the case of asphalt with high resins/asphaltenes ratio which leads to high viscous behaviour, higher softening points, and lower penetrations.

Resins are an intermediate weight, semisolid fraction formed of aromatic rings with side chains. Also, resins are polar molecules that act as peptizing agents to prevent asphaltenes molecules from coagulating. The lightest molecular weight materials are the nonpolar oils. Oils generally have a high proportion of chains as compared to the number of rings. From the literature, the resins and oils are referred to collectively as maltenes. In general, asphaltenes produce the bulk of the bitumen while resins contribute to adhesion and ductility and oils influence flow and viscosity properties [47]. According to the microstructure and the colloidal system, asphaltenes are diffused into an oily matrix of maltenes, encased by a shell of resins whereby its thickness varies with the temperature that is being tested [48]. Thus, bitumen composition and temperatures are strongly dependent on the mechanical properties and microstructure of bitumen and on the degree of aromatisation of the maltenes and the concentration of asphaltenes [48, 49].

#### 5.1.2. Asphalt Polarity and Morphology

**Figure 7:**
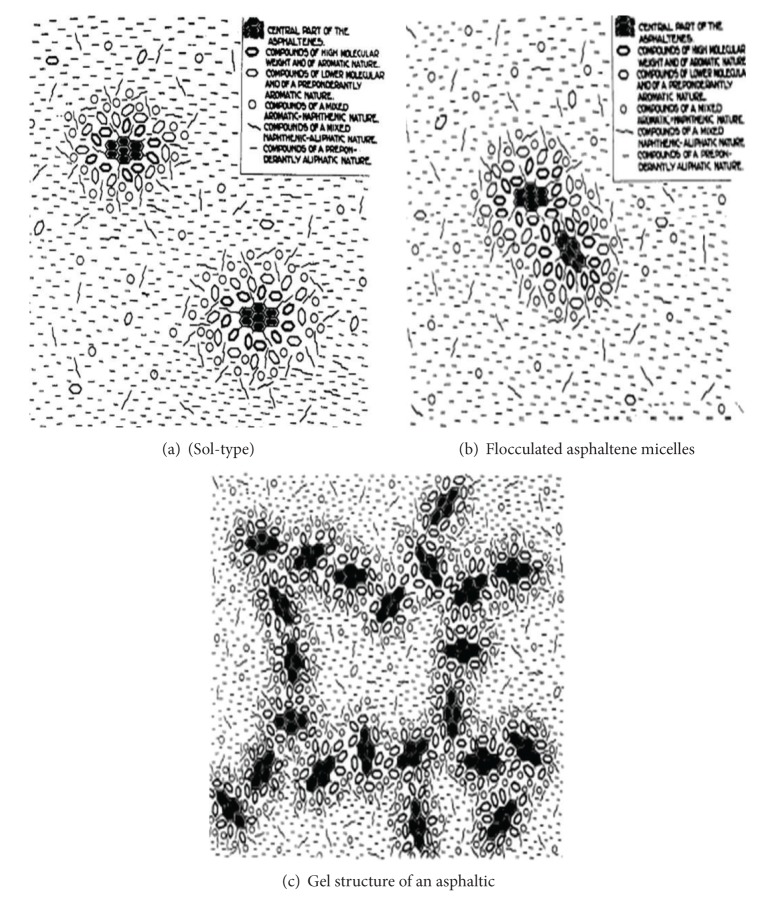
Asphalt colloidal model [51].

Asphalt has another important property which is polarity, which is the separation of charge within a molecule. Polarity is an important factor system because it refers to molecules managing themselves into preferred orientations. According to Robertson [50], most of the naturally occurring heteroatoms, nitrogen, sulfur, oxygen, and metals are strongly dependent on polarity within these molecules. Also, oxidation products upon aging are polar and further contribute to the polarity of the entire system. The physicochemical properties have an obvious significant effect on asphalt and each reflects the nature of the crude oil used to prepare it. Pfeiffer and Saal [51] suggested that asphalt cement dispersed phases are composed of an aromatic core surrounded by layers of less aromatic molecules and dispersed in a relatively aliphatic solvent phase. However, they do not point out that there are distinct boundaries between dispersed and solvent phases, as found in soap micelles. However, they suggest that it ranges from low to high aromaticity, that is, from the solvent phase to the centres of the entities constituting the dispersed phase as shown in Figure 7.

According to Robertson [50] the most consistent description, or model, of petroleum asphalt polarity is as follows. Asphalt cement is a collection of polar and nonpolar molecules: (i) the polar molecules are associated strongly to form organised structures and represent a more stable thermodynamic state. (ii) The nonpolar model has the ability to dissociate the organised structure, but again there are possible variations from asphalt sources and its viscous behaviours are highly dependent on the temperature.

**Figure 8:**
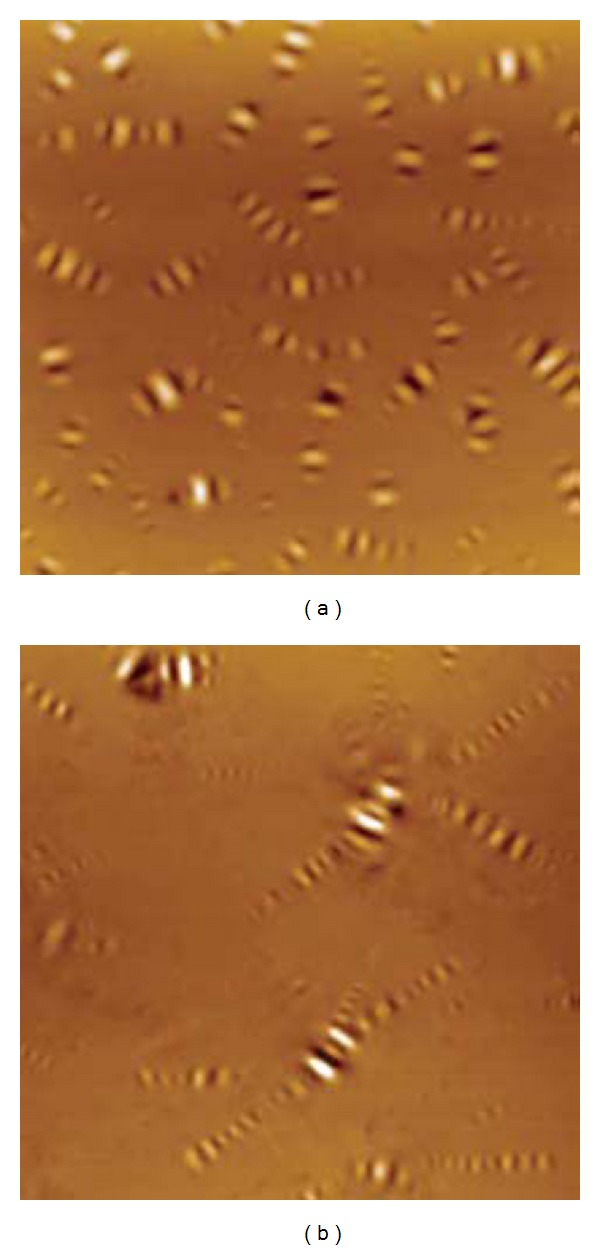
Topographic AFM images of two types of asphalt [52].

Using current day technology, the morphology of asphalt has been studied in order to verify the asphalt structure. Thus, Figure 8 presents the topographic atomic force microscopy (AFM) images of two different grades of asphalt cement, showing a flat background where another phase is dispersed [52].

In the image of the left side of Figure 8, the dispersed phase displays a range of pale and dark lines frequently regarded as “bees” or “bee structures.” However with the image on the right side where the bee-like structures are not independent of one another, they are substituted by “multiarm star-shapes” [52]. A dispersed phase, with a “bee-like” appearance as presented in Figure 8, is attributed to asphaltenes, which is also supported by Pauli et al. [53]. However, no correlation was found between the atomic force microscopy morphology and the composition made up of asphaltenes, polar aromatics, naphthene aromatics and saturates [52].

### 5.2. Crumb Rubber Properties

The use of rubber crumbs instead of polymer depends on the desired properties of the modified bitumen for a particular application. However, the choice is also determined to a certain extent by the cost of modification and the availability of the modifier [2]. The required properties are preferably obtained with minimal cost. The growth of vehicle productions year by year has generated wasted tyres. Due to limitation of disposal area and environmental problem the recycling of these vehicles tyres as industrial wastes has been encouraged, and the production of rubber crumbs from it has found it suitable for use as modifier into bitumen. Also it offers other benefits such as using with less complicated blending equipment, and minimal requirement to modify the asphalt. Comparing the use of polymer as a modifier, taking the two fundamental points cited above, the cost using polymer is much higher than using rubber crumbs and its availability is less compared to rubber crumbs. Even though the properties of using polymers may be better, they are comparable to those of rubberised asphalt.

#### 5.2.1. Crumb Rubber Constitutions and Concentration

Crumb rubber or waste tyre rubber, is a blend of synthetic rubber natural rubber, carbon black, antioxidants, fillers, and extender type of oils which are soluble in hot paving grade. Asphalt rubber is obtained by the incorporation of crumb rubber from ground tyres in asphalt binder at certain conditions of time and temperature using either dry process (method that adds granulated or crumb rubber modifier (CRM) from scrap tires as a substitute for a percentage of the aggregate in the asphalt concrete mixture, not as part of the asphalt binder) or wet processes (method of modifying the asphalt binder with CRM from scrap tires before the binder is added to form the asphalt concrete mixture). There are two different methods in the use of tyre rubber in asphalt binders; first one is by dissolving crumb rubber in the asphalt as binder modifier. Second one is by substituting a portion of fine aggregates with ground rubber that does not completely react with bitumen [22].

According to laboratory binder tests [10–12], it is clear that rubber crumb content played a main role in influencing significantly the performance and rheological properties of rubberised bitumen binders. It could enhance the performance properties of asphalt pavement resistance against deformation during construction and road services. The increase in rubber crumb content was from 4 to 20% thus indicating a liner increase in softening point, ductility, elastic recovery, viscosity, complex shear modulus, and rutting factor. This phenomenon could be explained by the absorption of rubber particles with lighter fraction oil of bitumen, leading to increase in rubber particles during swelling during the blending process. The increase in rubber content by 16% and 20% showed a corresponding increase in Brookfield viscosity value that is higher than SHRP specification limits (3 Pa). These make the two stated percentages unacceptable for field construction during asphalt pavement mixture construction.

Regarding the low temperature performance, an investigation with 18–22% of rubber content showed change that was little significance within this range in affecting the tensile and fracture performance of the bitumen compared to varying the binder content between 6 and 9% by bitumen weight [22, 54]. A study by Khalid [55] found that higher binder content led to longer fatigue life of rubberised bitumen mixture and better resistance to rutting as well as results showing good resistance to fracture and fatigue cracking. Liu et al. [56] found that content of crumb rubber is the most significant affecting factor followed by crumb rubber type and lastly the size of the particle.

#### 5.2.2. Crumb Rubber Grinding Process and Particle Size

**Figure 9:**
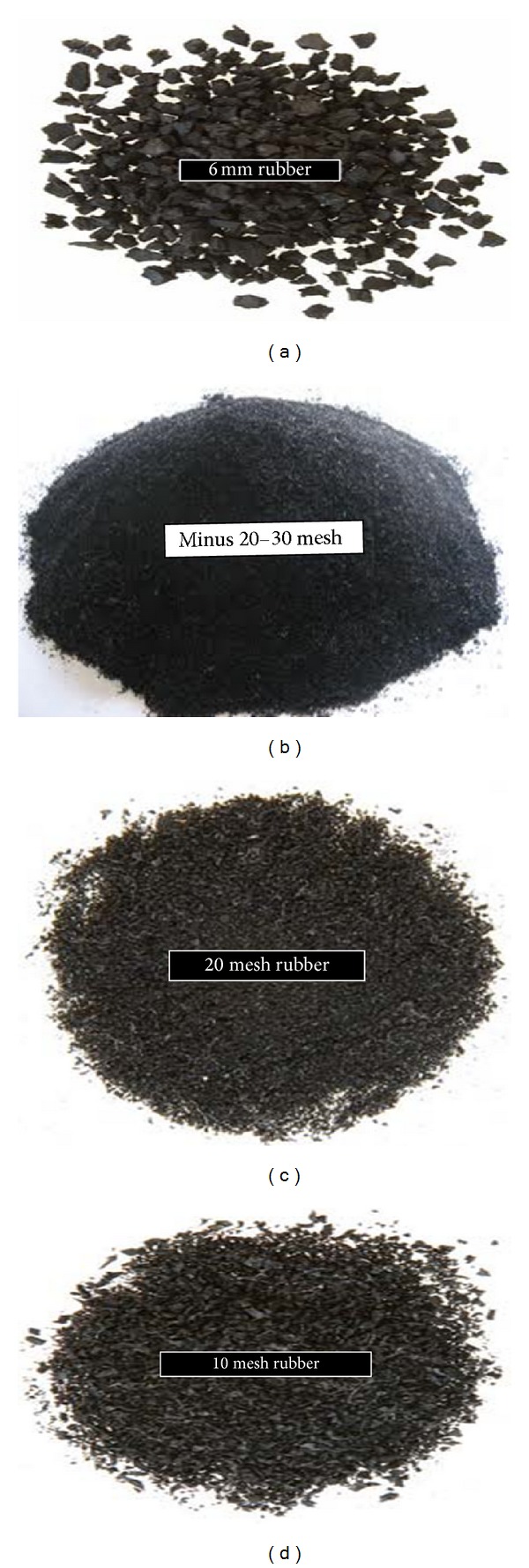
Different type of crumb rubber based on particle size.

Crumb rubber is made by shredding scrap tyre, which is a particular material free of fibre and steel. The rubber particle is graded and found in many sizes and shapes as shown in Figure 9. To produce crumb rubber, initially it is important to reduce the size of the tyres. There are two techniques to produce crumb rubber: ambient grinding and the cryogenic process [57]. In the crumb rubber market, there are three main classes based on particle size:

- type 1 or grade A: 10-mesh coarse crumb rubber,
- type 2 or grade B: 14- to 20-mesh crumb rubber,
- type 3: 30-mesh crumb rubber.

Mesh size designation indicates the first sieve with an upper range specification between 5% and 10% of material retained. Ambient grinding process can be divided into two methods: granulation and cracker mills. Ambient describes the temperature when the waste tyres rubber size is reduced. The material is loaded inside the crack mill or granulator at ambient temperature. Whereas, cryogenic tyre grinding consists of freezing the scrap tyre rubber using liquid nitrogen until it becomes brittle and then cracking the frozen rubber into smaller particles with a hammer mill. The resulting material is composed of smooth, clean, flat particles. The high cost of this process is considered a disadvantage due to the added cost of liquid nitrogen [3].

The particles size disruption of crumb rubber influenced the physical properties of asphalt-rubber blend. In general, small difference in the particles size has no significant effects on blend properties. However, the crumb rubber size can certainly make a big difference. A study [58] reported that the particle size effects of CRM on high temperature properties of rubberised bitumen binders was an influential factor on viscoelastic properties. Also, coarser rubber produced a modified binder with high shear modules and an increased content of the crumb rubber decreased the creep stiffness which in tandem displayed better thermal cracking resistance.

In summary, the primary mechanism of the interaction is swelling of the rubber particles caused by the absorption of light fractions into these particles and stiffening of the residual binder phase [58–61]. The rubber particles are constricted in their movement into the binder matrix to move about due to the swelling process which limits the free space between the rubber particles. Compared to the coarser particles, the finer particles swell easily thus developing higher binder modification [58, 59]. The swelling capacity of rubber particle is linked to the penetration grade of the binder, crude source, and the nature of the crumb rubber modifier [60].

### 5.3. Interaction Process Variables

Interaction process variables consist of curing profile of temperature and duration and mixing shear energy [12, 58, 59, 62]. A study [63] studied the effect of the mixing types on the properties of rubberised asphalt. An ordinary propeller-type mixer and the high speed shear mixer were used. The study indicated that the resultant binder produced using the high-speed shear mixer appears to have slightly superior properties as compared to that produced using the propeller-type mixer. It showed that the viscosity and softening point of the rubberised asphalt produced using the high-speed shear mixer produced higher agitation level and shearing action that can chop the swollen rubber particles within a certain volume of binder. Thus, the absorbent of the lighter oily fraction was increased due to the large amount of the small rubber particles. A study by Thodesen et al. [64] indicated that processing procedure and tyre type play an important role in the determination of rubberised bitumen viscosity. Interaction between crumb rubber and bitumen binders is referred to as a physical interaction where the crumb rubber through diffusion absorbs the aromatic fraction of the bitumen binders which leads to swelling of the crumb rubber particles. This particle swelling compounded with reduction in the oily fraction of the binder results in increased viscosity in the rubberised bitumen binder. In general, the bitumen binder and ground tyre rubber are mixed together and blended at elevated temperatures for differing periods of time prior to using them as a paving binder. These two factors work together to evaluate the performance properties of rubberised bitumen binder through blending process of asphalt rubber interaction. This variation in mixing time and temperature results due to the normal activities which are related to bitumen paving construction [2]. Nevertheless, the consistency of asphalt rubber can be affected by the time and temperature used to combine the components and thus must be cautiously used for its optimum potential to be achieved. The increase in blending time showed insignificant difference on rubberised asphalt properties in the case of 30 and 60 minutes, whereas the increase in blending temperature corresponded to the increase in Brookfield viscosity, softening point, ductility, elastic recovery, and complex shear modulus [10–12]. Several studies [62, 65–67] showed that longer reaction time for production of the asphalt rubber apparently caused increased viscosity due to the increased rubber mass through binder absorption. On the other hand [12, 61, 68–70] reported that the reaction time has no significant effect on the selection of the optimal binder content. In addition, there was no difference in the molecular size variation between the control binder and the asphalt rubber binders. Also, blending time had insignificant difference on asphalt rubber physical and rheological properties and a rather slight influence on the performance properties of rubberised asphalt.

### 5.4. Elasticity of Tyre Rubber

The main characteristics of rubber is its property of high elasticity which allows it to undergo large deformations from which almost complete, instantaneous recovery is achieved when the load is removed [71]. This property of high elasticity derives from the molecular structure of rubber. Rubber belongs to the class of materials known as polymers and is also referred to as an elastomer. The properties of an elastomer rubber are as follows: (a) the molecules are very long and are able to rotate freely about the bonds joining neighbouring molecular units. (b) The molecules are joined, either chemically or mechanically, at a number of sites to form a three-dimensional network. These joints are termed cross-linked. (c) Apart from being cross-linked, the molecules are able to move freely past one another; that is, the Van der Waal's forces are small.

Similar to asphalt, rubber is a thermoplastic, visco-elastic material, whose deformation response under load is related to both temperature and rate of strain. However, the deformation of rubber is relatively incentive to temperature change where at both low rates of strain and at temperature well above the ambient, the material remains elastic. The wider range of elastic behaviour of rubber compared to that of bitumen largely results from the cross-linking of the long rubber molecules. Rubber is also much more ductile than bitumen at low temperatures and high loading rates [2, 3].

## 6. Rheological and Physical Characteristics of Asphalt Rubber

### 6.1. Temperature Susceptibility (Newtonian Behaviour)

The temperature susceptibility was defined as a ratio of Newtonian viscosities at 25°C and 60°C [72]. The binder content in the asphaltic mix is usually less than 7% but it plays a very significant role in the overall properties of the composite material. It strongly affects both the load spreading capability and resistance to distortion under heavy traffic. The deformation response of a binder in a mix under load depends on its temperature sensitivity; the temperature range is subjected to rate of strain and the geometry of binder between the aggregate particles. Therefore, it is logical to use a binder with lower temperature susceptibility, particularly when the range of working temperatures is very high [2]. The concept of the penetration index (PI) was introduced by Pfeiffer and Van Doormaal [73] to measure both the binder's temperature susceptibility and, in particular, its rheological type in terms of deviation from Newtonian behaviour. PI is obtained from the relationship

(1) $$\begin{array}{c} \frac{d\log\text{pen}}{dT}=\frac{\left(20-\text{PI}/10+\text{PI}\right)}{50}. \end{array}$$

Normal road paving asphalt has a PI value between −1 and +1. Asphalt with PI below −2 is substantially Newtonian and characterised by brittleness at low temperature. Asphalt with PI above +2 is far less temperature susceptible, is less brittle at low temperature, indicates marked time dependent elastic properties, and shows deviations from Newtonian behaviour, especially at large strain rates [74]. The coefficients of temperature susceptibility (CTS) based on viscosity measurements in the temperature range 60°–80°C were used to assess the behaviour of rubberised asphalt binder with temperature. CTS is obtains from (2) as shown in:

(2) $$\begin{array}{c} \text{CTS}=\frac{\log\left(\log\eta_{T_{1}}/\log\eta_{T_{2}}\right)}{\log\left(T_{2}/T_{1}\right)}, \end{array}$$

where *T* is Temp °F and *η*_*T*_1__ and *η*_*T*_2__ are viscosities measured at temperatures *T*_1_ and *T*_2_.

In 1984, a research study found that 4% rubber is effective in reducing the temperature susceptibility of virgin binders by a factor of at least two. Hence, asphalt rubber is more resistant to rapid changes in temperature [74].

Mashaan and Karim [12] investigated a good correlation between temperature susceptibility and rheological properties of crumb rubber modified asphalt in term of elasticity and softening point data.

### 6.2. Viscoelastic Behaviour (Dynamic Shear)

**Figure 10:**
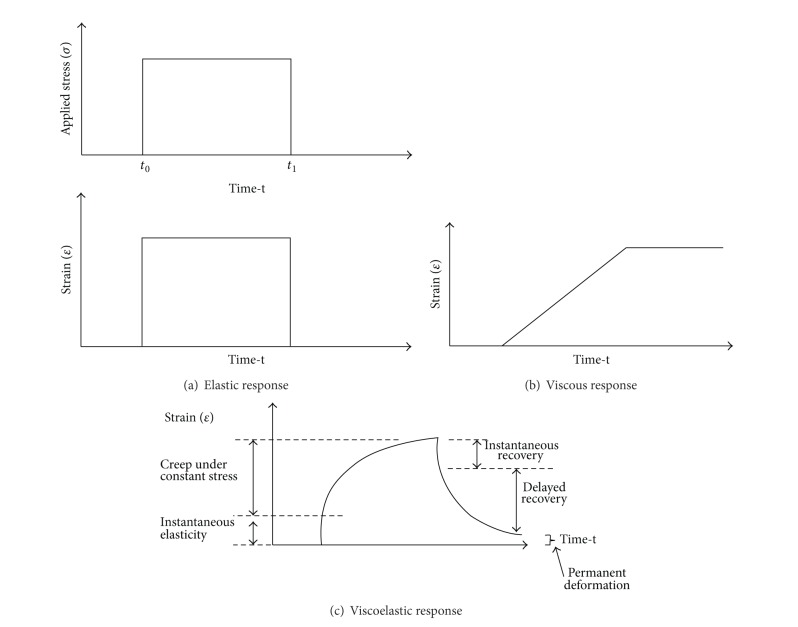
General behaviour of elastic, viscous, and viscoelastic material under constant stress loading [75].

Asphalt cement binders are referred to as viscoelastic materials because they exhibit combined behaviour (properties) of elastic and viscous material as presented in Figure 10(a) with the removal of the applied stress from the material; there is a complete recovery to the original position.  Figure 10(b) explains the behaviour of a viscous material in case the strain of the material increases through time under stable stress.  Figure 10(c) illustrates the behaviour of a viscoelastic material when stable stress increases the strain over a long period of time and when the applied stress is removed, the material loses its ability in attaining its original position resulting in permanent deformation. According to Van der Poel [75], generally the stiffness modulus of bitumen binders can be defined by

(3) $$\begin{array}{c} S\left(t\right)=\frac{\sigma }{\varepsilon \left(t\right)}, \end{array}$$

where, *S*(*t*) is dependent stiffness modulus (Pa), (*t*) is loading time (s), *σ* is the applied constant uniaxial stress (Pa), and *ε*(*t*) refers to uniaxial strain at time *t*, (m/m). Since asphalt is a viscoelastic material, its rheological properties are very sensitive to temperature as well as to the rate of loading. With respect to temperature, the most frequent problems of road pavement are rutting, fatigue cracking, and thermal cracking. Dynamic shear rheometer (DSR) was used to measure and determine the rheological properties of the asphalt binder at different stress/temperature sweep and various frequencies. DSR testing included parameters of complex shear modulus (G*), storage modulus (*G*′), loss modulus (*G*′′), and phase angle (*δ*). The formula to calculate the G*, *G*′, and *G*′′ as well as *δ* in (4), respectively, is demonstrated as follows:

(4) $$\begin{array}{c} G^{*}=\left(\frac{\tau }{\gamma }\right),\\ G^{\prime }=\cos\left(\delta \right)\left(\frac{\tau }{\gamma }\right),\\ G^{\prime \prime }=\sin\left(\delta \right)\left(\frac{\tau }{\gamma }\right),\\ \delta =\frac{G}{G^{\prime \prime }}, \end{array}$$

where G* is the complex shear modulus, *τ* is the shear stress, *γ* is the shear strain, *G*′ is the storage modulus, *G*′′ is the loss modulus, and *δ* is the phase angle.

Navarro et al. [40] studied the rheological characteristics of ground tire rubber-modified asphalt. The experiment was performed in a controlled-stress Haake RS150 rheometer. The study aimed at comparing the viscoelastic behaviour of five ground tyres rubber modified with unmodified asphalt and polymer-modified (SBS) asphalt. The study displayed that rubber-modified asphalt improved viscoelastic characteristics and therefore has higher viscosity than unmodified binders. Thus, the asphalt rubber is expected to better enhance resistance to permanent deformation or rutting and low temperature cracking. The study also found that the viscoelastic properties of rubber-modified asphalt with 9% weight are very similar to SBS-modified bitumen having 3% weight SBS at −10°C and 7% weight at 75°C.

Mashaan and Karim [12] investigated the rheological properties of asphalt rubber for various combination factors of crumb rubber content and blending conditions. The dynamic shear rheometer (DSR) test was conducted to evaluate the engineering properties of asphalt binder reinforced with crumb rubber at 76°C. Specification testing was performed at a test frequency of 10 rad/s which is equivalent to the car speed of 90 km/h. Test specimens of 1 mm thick by 25 mm in diameter were formed between parallel metal plates. The study displays increases in G*, *G*′, and *G*′′ and decrease in phase angle (*δ*). Thus, the modified asphalt became less susceptible to deformation after stress removals. The study also presented a considerable relationship between rheological parameters (G*, *G*′, *G*′′, and *δ*) and softening point in terms of predicting physical-mechanical properties regardless of blending conditions.

Natu and Tayebali [76] observed that the unmodified and crumb rubber modified binders with the same high temperature PG rating do not show similar viscoelastic behaviour over a range of frequencies. It was also concluded that the unmodified and crumb rubber modified mixtures containing binders with the same high temperature PG rating do not show similar viscoelastic behaviour over a range of frequencies. Mixtures containing the same PG rated binders performed similarly if their performance was evaluated at a frequency and temperature at which the binder high temperature PG rating was determined.

The loss tangent (Tan *δ*) of the binder was not observed to be directly related to the loss tangent of the mixture, since the loss tangent of the mixture was much lower, perhaps due to the aggregate effects, than the loss tangent of the binder. It was also noted that the loss tangent of the mixture increased when the temperature was decreased. A similar observation was also made for the influence of frequency. With increasing frequency, the loss tangent increased to a peak value and then decreased with further increase in frequency. The loss tangent of the binder increased appreciably when the temperature was increased [2]. Mixture stiffness by itself does not appear to provide a measure for assessing the propensity for rutting in mixtures containing modified binders. Higher dynamic modulus (G*) are not necessarily associated with lower permanent deformation. With respect to binder type, the dynamic modulus is lower for the mixtures containing modified binders as compared to the mixture containing conventional binder [2].

At high service temperatures, rutting resistance tests were measured as a function of some binder parameters (viscosity, ductility recovery, nonrecoverable creep compliance, complex shear modulus G*, and parameter specified by SHRP G*/sin⁡*δ*). It was concluded that, of the parameters considered, for this range of binders, only the SHRP G*/sin⁡*δ* gives the most reliable prediction of rut resistance. The SHRP recommended frequency (1.6 Hz) was found to correspond closely to the frequency of the wheel tracking test used for rutting resistance experiments. This parameter includes both a measure of the stiffness of the binder (its ability to resist deformation when a load is applied) and its ability to recover any deformation when the load is removed. The frequency selected for the binder measurements has been found to have a significant impact on the quality of the correlation obtained and should be as possible to the frequency of loading applied to the mix [2]. At intermediate pavement service temperatures a reasonable correlation was found between one aspect of mix fatigue performance (*ε*) and the binder loss modulus (G*sin⁡*δ*), again measured under the same temperature and loading as the mix testing. However, above certain binder stiffness, the variation in measured fatigue life was small, due to machine compliance becoming significant at high mix stiffness. It is unlikely that binder rheology alone will be sufficient to accurately predict and explain mix fatigue life [2].

### 6.3. Viscosity Property (Flow Resistance)

Viscosity refers to the fluid property of the asphalt cement and it is a gauge of flow-resistance. At the application temperature, viscosity greatly influences the potential of the resulting paving mixes. During compaction or mixing, low viscosity has been observed to resulting in lower stability values and better workability of asphalt mixture.

Nair et al. [77] used a Haake rotational viscometer to measure the viscosity of the soft asphalt samples while the viscosity of the blown asphalt samples is measured on a capillary rheometer. The tests were conducted to study the flow behaviour on the modification of asphalt with liquid natural rubber (LNR). The findings are as follows; for soft asphalt, the temperature dependence on viscosity is prominent up to 100°C and subsequently marginal. The addition of 20% LNR results in the maximum viscosity. Activation energy of flow of soft bitumen increased, while that of blown asphalt decreased on addition of LNR.

Zaman et al. [78] found that the viscosity of asphalt cement increases with the addition of rubber, and rubber-modified asphalt-cement samples show a more uniform and higher resistance against loading as the amount of rubber increased. The degrees of shear-thickening and shear-thinning behaviour decreased by increasing the amounts of rubber in asphalt cement. The liner dynamic viscosity was increased by increasing the amount of rubber in asphalt cement. Piggott et al. [79] mentioned that the vulcanized rubber had a large effect on the viscosity of the asphalt cement. The viscosity, measured at 95°C, increased by a factor of more than 20 when 30% vulcanized rubber was added to the mixture. In contrast, the devulcanized rubber had only a very small effect. The viscosity test also showed that there is no danger of gel formation when rubber is mixed with hot asphalt cement.

### 6.4. Physical Behaviour and Stiffness Performance

Mahrez [2] investigated the properties of asphalt rubber binder prepared by physical blending of asphalt 80/100 penetration grade with different crumb rubber content and various aging phases. The results of penetration values decreased over the aging as well as before aging by increasing the rubber content in the mix. Also, the modified binders showed lower penetration values than unmodified binders. Another study [80] on penetration change was conducted using asphalt 80/100 and 70/100 penetration grade mixes with different crumb rubber percentage. The results showed a significant decrease in the penetration values of modified binder due to high crumb rubber content in the binders. According to Jensen and Abdelrahman [30], elastic recovery property is very important in both fatigue and rutting resistance selection and evaluation. Elastic recovery is a property that indicates the quality of polymer components in asphalt binders. Oliver [81] concluded from his study that the elastic recovery of asphalt rubber binders leads to an increase as the rubber particle size decreases. It was found that rubber types could affect the force ductility properties at 4°C [82]. Asphalt rubber modification resulted in a better rutting resistance and higher ductility. However, the modified binder was susceptible to decomposition and oxygen absorption. There were problems of low compatibility because of the high molecular weight. Furthermore, it was found that recycled tyre rubber decreases reflective cracking, which in turn increases durability. During compaction or mixing, low viscosity has been observed to resulting in lower stability values. Softening point refers to the temperature at which the asphalt attains a particular degree of softening [3]. Mahrez and Rehan [83] claimed that there is a consistent relationship between viscosity and softening point at different aging phases of asphalt rubber binder. Also, it is reported that the higher crumb rubber content leads to higher viscosity and softening point.

Mashaan and Karim [12] reported that the softening point value increases as crumb tuber content increases in the mix. The increase of rubber content in the mix could be corelated to the increase in the asphaltenes/resins ratio which probably enhanced the stiffening properties, making the modified binder less susceptible to temperature changes. According to Liu et al. [56], the main factor in the increase in softening point can be attributed to crumb rubber content, regardless of type and size. The increase in softening point led to a stiff binder that has the ability to enhance its recovery after elastic deformation. According to Mashaan et al. [11], the rubberised asphalt binder was evaluated in terms of binder elasticity and rutting resistance at high temperature. The higher crumb rubber content appears to dramatically increase the elastic recovery and ductility. According to a study [71], the ductility test conducted at low temperature was found to be a useful indicator of brittle behaviour of bitumen. Latex contents in the range from 3 to 5% were found to result in nonbrittle behaviour in the ductility test at 5°C whereas the unmodified bitumen failed by brittle fracture in the same test. Nair et al. [77] found that the ductility decreased in the case of soft bitumen with increasing concentration of liquid natural rubber while some improvement was noticed in the case of blown bitumen at 10% loading. The ductility is measured at 27°C and pulled apart at a rate of 50 mm/min. Modified bitumen binders showed a significant enhancement on the elastic recovery, and, in contrast, the ductility decreased with respect to unmodified binders [84].

## 7. Durability and Aging of Asphalt Rubber

In the paving design mixture, the general practice is to arrive at a balanced design among a number of desirable mix properties, one of which is durability. Durability is the degree of resistance to change in physicochemical properties of pavement surface materials with time under the action of weather and traffic. The life of a road surfacing will depend primarily on the performance of the binder provider, the mix design, and construction techniques [2]. Asphalt hardening can lead to cracking and disintegration of the pavement surface. The rate of hardening is a good indicator of the relative durability. Many factors might contribute to this hardening of the asphalt cement such as oxidation, volatilisation, polymerization, and thixotropy. This is because asphalt is an organic compound, capable of reacting with oxygen found in the environment. The asphalt composite changes with the reaction of oxidation developing a rather brittle structure. This reaction is referred to as age hardening or oxidative hardening [85]. Volatilisation occurs when the lighter components of the asphalt evaporate. In general, this is related to elevated temperatures that are found firstly during the hot mix asphalt production process. Polymerisationis the means by which resins are assumed to combine into asphaltenes, resulting in an increase in the brittleness of the asphalt along with a tendency toward non-Newtonian behaviour. At the end of the reaction, thixotropy, or an increase in viscosity over time, also contributes to the aging phenomenon in asphalt [85]. However, the most important factors in the aging process of asphalt binder seem to be oxidation and volatilisation. The occurrence of steric hardening and the time-dependent reversible molecular association have affected the binder properties but this is not considered as aging. Steric hardening is only a factor at intermediate temperatures; at high temperatures excess kinetic energy in the system prevents the association and at low temperatures the rate of association is found to be slower due to the binder's high viscosity [85].

Bahia and Anderson [86] studied the mechanism by which binder properties may change at low temperature. This mechanism which is called physical hardening occurs at temperatures next to or lower than the glass transition temperature and causes significant hardening of the asphalt binder. The rate and magnitude of the hardening phenomena have been observed to increase with decreasing temperatures and is reported to be similar to the phenomena called physical aging on amorphous solids [87]. The physical hardening can be explained using the free volume theory which introduced the relationship between temperature and molecular mobility. The free volume theory includes the molecular mobility dependent on the equivalent volume of molecules present per unit of free space or free volume. Based on the free volume theory, when amorphous material is cooled from a temperature above its glass transition temperature, molecular adjustments and the collapse of free volume rapidly show a drop in temperature. At that temperature, the structural state of the material is frozen-in and deviates from thermal equilibrium due to the continuous drop in kinetic energy. Hence, it has been postulated that, in order for physical hardening to happen in binders, temperatures must be higher than the glass transition temperature.

Many durability tests are based on the evaluation of resistance to asphalt hardening. Mahrez and Rehan [83] investigated ageing effects on viscoelastic properties of rubberised asphalt using the Dynamic Shear Rheometer (DSR). The binders were aged with the Thin Film Oven Test (TFOT), the Rolling Film Oven Test (RFOT), and the Pressure Ageing Vessel (PAV). This research found that ageing influences the rheology of rubberised asphalt. The mechanical properties of aged binder improved by increased complex modulus and decreased phase angle. The aged samples were characterized by higher stiffness and elasticity, due to an increase in the elastic (storage) modulus, *G*′. The high value of *G*′ is an advantage since it improved further the rutting resistance during service. Natu and Tayebali [76] carried out comprehensive research study which evaluated high temperature performance characteristics of unmodified and crumb rubber modified asphalt binders and mixtures. The research showed that the effect of RFTO aging on binder rutting factor was increased at low frequencies and/or high temperatures. The improvement in the rutting factor diminished with an increase in frequency, and at very high frequencies (low temperatures) the rutting factors for unaged and RFTO aged binders were nearly the same. The increase in binder rutting factor of crumb rubber modified asphalt binders at low frequencies suggested that the binder resistance to permanent deformation has improved. Ali et al. [88] studied the influence of the physical and rheological properties of aged rubberised asphalt. The results indicate that the use of rubberised binder reduces the aging effect on physical and rheological properties of the modified binder as illustrated through lower aging index of viscosity (AIV), lower aging index of G*/sin⁡*δ*, lower softening point increment (Δ*S*), less penetration aging ratio (PAR), and an increase in Tan *δ* with crumb rubber modifier content increasing, indicating that the crumb rubber might improve the aging resistance of rubberised binder.

## 8. Failure of Road Pavement: Cracking and Permanent Deformation

Two kinds of loading are of specific importance in tandem with the performance of bituminous surfacing. One is due to vehicles loads passing over the road surfacing, while the second is due to thermal contraction in relation to temperature changes [81]. Vehicle loading can lead to distress at either end of the range of pavement surface temperatures. At increased pavement temperatures, the binder can be extremely fluid and probably will not resist the plucking and shearing action of vehicle tyres. At low pavement temperatures, the binder can be so hard (particularly after a long period of service) that vehicle loading causes brittle fracture of the binder films. The explanation to this phenomenon is thought to be due to the theory of “Normal Stresses” (Wiesenberger effect) which applies to viscoelastic material such as a bitumen/scrap rubber mixture. This theory covers normal stress differences, which are forces that develop normally (that is perpendicular) to the direction of shear [81].

According to the theory, a viscoelastic material forced through an open tube expands normally to the axis of the tube on leaving the tube. In a cracked pavement, the vertical loads are applied by the vehicle wheels which compel the bitumen binder to expand normally to the applied vertical load (horizontally) and thus fill up the cracks. Another reason is that, if this bitumen's mixture is stirred while being hot with a stick in a container, the material will climb up the stick, rather than forming a vortex as found in Newtonian type fluids [81].

### 8.1. Correlation between Rheological Properties of Asphalt Binder and Performance of Asphalt Mixture

As extensive research program conducted [89] to investigate the benefits of using fundamental binder rheological measurements to predict asphalt pavement performance included

- pavement deformation (rutting) at high service temperatures,
- fatigue at intermediate service temperatures,
- brittle fracture at low service temperatures.

At high service temperatures, rutting resistance tests were measured as a function of some binder parameters (viscosity, ductility recovery, nonrecoverable creep compliance, complex shear modulus G*, and parameter specified by SHRP G*/sin⁡*δ*). It was concluded from the parameters considered that, for this range of binders, only the SHRP G*/sin⁡*δ* gives the most reliable prediction of rut resistance. The SHRP recommended frequency (1.6 Hz) was found to correspond closely to the frequency of the wheel tracking test used for rutting resistance experiments. This parameter includes both a measure of the stiffness of the binder (its ability to resist deformation when a load is applied) and its ability to recover any deformation with the removal of the load. The frequency selected for the binder measurements was to have a significant impact on the quality of the correlation obtained and should be maintained close to the frequency of loading applied to the mix [89].

At intermediate pavement service temperatures a reasonable correlation was found between one aspect of mix fatigue performance (*ε*) and the binder loss modulus (G*sin⁡*δ*), again measured under the same temperature and loading as the mix testing. However, above certain binder stiffness, due to machine compliance being significant at high mix stiffness the variation in measured fatigue life was minimal. Binder rheology alone is not adequate to accurately predict and explain mix fatigue life. At low pavement service temperatures a binder limiting stiffness temperature (LST) in this case based on G* = 300 Mpa at 1000 s provides a good indicator of the fracture temperature of the mix [89].

### 8.2. Fatigue Resistance of Asphalt Rubber

Bahia and Davies [90] used the rheological properties as indicators for the pavement performance. At high temperature the rheological properties were related to the rutting performance of pavements. The rheology at intermediate temperatures had an impact on the fatigue cracking of pavements. The low temperature properties of the binder are related to the low-temperature thermal cracking of the pavement. Temperature additionally is a vital factor that is correlated with the rate of loading. At elevated temperatures, or slow rates of loading, bitumen becomes a viscous material.

However, at decreased temperatures or higher rates of loading, bitumen then becomes a highly elastic material. In fact at intermediate temperatures, bitumen has two different characteristics, that is, an elastic solid and a viscous fluid [75].

Aflaki and Memarzadeh [91] investigated the effects of rheological properties of crumb rubber on fatigue cracking at low and intermediate temperatures using different shear methods. The results showed that the high shear blending has more effect on improvement at low temperatures than the low shear blend.

**Figure 11:**
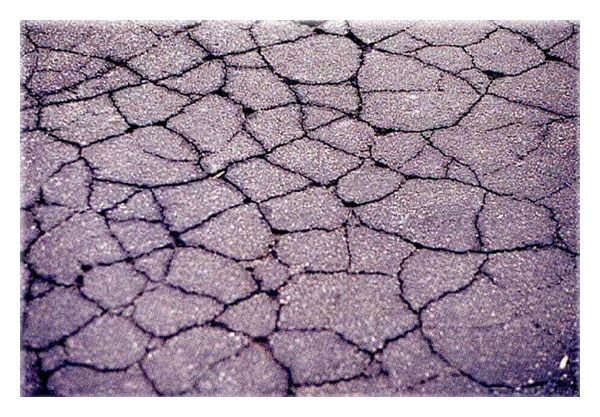
Fatigue cracking.

Bahia and Anderson [92] presented a description of the purpose and scope of the dynamic shear rheometer test. The dynamic shear rheometer (DSR) was used to characterise the viscoelastic behaviour of bituminous material at intermediate and high service temperatures. Stress-strain behaviour defines the response of materials to load. Asphalt binders exhibit aspects of both elastic and viscous behaviours; hence they are referred to as viscoelastic materials. Bahia and Anderson [86] conducted a time sweep test using dynamic shear rheometer. The test is a simple method of applying repeated cycles of stress or strain loading at selected temperatures and loading frequency. The initial data under repeated loading in shear showed that the time sweeps are effective in measuring binder damage behaviour. One of the advantages of the time sweep test is that it can be used to calculate fatigue life of asphalt binder based on dissipated energy approaches. Fatigue is one of most important distresses in asphalt pavement structure due to repeated load of heavy traffic services which occur at intermediate and low temperatures as shown in Figure 11. The use of crumb rubber modified with bitumen binder seems to enhance the fatigue resistance, as illustrated in a number of studies [3, 6, 18, 88, 91, 93–95]. The improved performance of bitumen-rubber pavements compared with conventional bitumen pavements has partly resulted from improved rheological properties of the rubberised bitumen binder.

Cracking is normally considered to be low temperature phenomena while permanent deformation is considered the predominant mode of failure at elevated temperatures. Cracking is mainly categorised into thermal cracking and load-associated fatigue cracking. Large temperature changes that occur in pavement usually result in thermal cracking. This type of failure occurs when the thermally induced tensile stress, together with stresses caused by traffic, exceeds the tensile strength of the materials. It is often characterised by transverse cracking along the highway at certain intervals. Load-associated fatigue cracking is the phenomenon of fracture as a result of repeated or fluctuated stresses brought about by traffic loading. Traffic loads can cause a pavement structure to flex and the maximum tensile strain will occur at the base of the bituminous layer. If this structure is inadequate for the imposed loading conditions, the tensile strength of the materials will be exceeded and cracks are likely to initiate, which will be manifested as cracks on the surface of the pavement [9].

This resistance of bituminous mixtures to cracking is essentially dependent upon its tensile strength and extensibility characteristics. These can be achieved by simply increasing the bitumen content of the mix. However such an attempt may have an adverse effect on the mix stability. The use of softer bitumen can also improve the mix flexibility but this can only be achieved at the expense of the tensile strength and stability of the mix [9].

**Figure 12:**
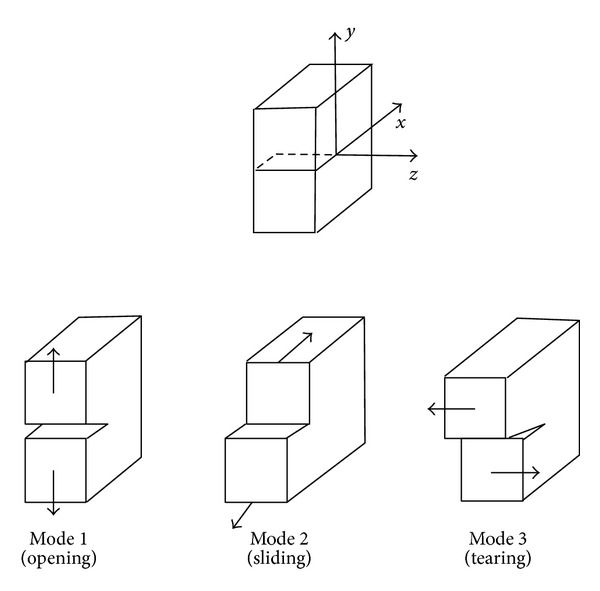
Modes of crack displacement [79].

In the fracture mechanics approach, fatigue cracking process of pavement systems is considered to develop in two distinct phases involving different mechanisms. These phases consist of crack initiation and crack propagation before the material experiences failure or rupture. Crack initiation can be described as a combination of microcracks within the mix forming a macrocrack as a result of repeated tensile strains. This occurrence usually creates gradual weakening of the structural component [96]. These microcracks become more visible as the stress concentrations at the tip of the crack increase and cause further crack propagation. Crack propagation is the growth of the macrocrack through the material under additional application of tensile strains. The actual mechanism of crack initiation and propagation involves fracture of the overlay when the tensile stresses exceed the tensile strength under the particular conditions [9]. For an accurate determination of the crack propagation, the magnitude of the stress intensity factors over the overlay thickness should be available for each fracture mode. In general, the mechanisms of cracking propagation can follow one or more of the three fracture modes which are directly related to the type of displacement induced [97]. This is shown in Figure 12.

- Mode I loading (opening mode) results from load applied normally to the crack plane (normal tension). This mode is associated with traffic loading and in the case of thermally induced displacement.
- Mode II loading (sliding mode) results from in plane/normal shear loading, which leads to crack faces sliding against each other normal to the leading edge of the crack. This mode is usually associated with traffic loading or differential volume changes.
- Mode III loading (tearing mode) results from out of plane shear (parallel shear) loading, which causes sliding of the crack faces parallel to the crack loading edge. This mode may occur under lateral displacement due to instability, if the crack plane is not normal to the direction of traffic.

### 8.3. Rutting Resistance of Asphalt Rubber

There are various laboratory methods for studying distortion or rutting. The TRRL Wheel Tracking Test appears to be the most suitable in stimulating the field conditions as closely as possible. The test was conducted for 24 hours in temperature controlled cabinet at 60°C. From the indents made on the slab, the depth of tracking was recorded at the mid-point of its length. After about 6 hours, a steady state of tracking was observed. From the deformation/time curve, the rate of increase in track depth is determined in mm per hour once the steady state is reached [19].

According to Shin et al. [98], addition of crumb rubber and SBR increases the rutting resistance of asphalt paving mixtures. The results from laboratory study showed that the CR-modified and SBR-modified asphalt had higher stiffness at 60°C than the modified mixtures. The modified asphalt mixtures also had higher gyratory shear strengths and lower rut depths in the Loaded Wheel tests than the unmodified mixtures.

Tayfur et al. [99] claimed that, after the initial densification, the permanent deformation of the bituminous mixture happens due to shear loads which take place close to the pavement surface which in fact is the contact area between the tyre and the pavement. These efforts increase without the volume variations in the bituminous mixture. They are the primary mechanisms in the development of rutting during the life span of the pavement design.

**Figure 13:**
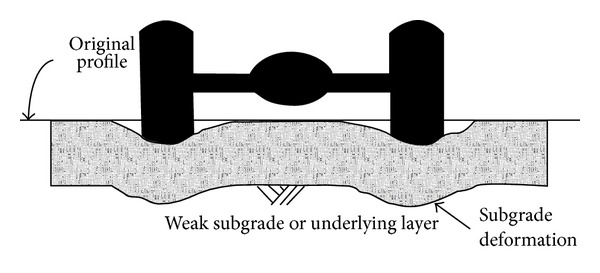
Consolidations rutting of flexible pavement [101].

**Figure 14:**
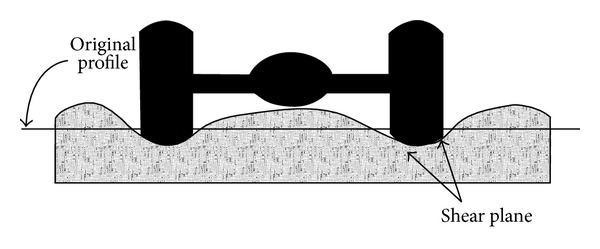
Instability rutting of flexible pavement [101].

Increased permanent deformation or rutting has been related to the increase in truck tyre pressures, axle loads, and volume of traffic [100]. A study [2] claimed that the use of rubberised bitumen binder has a significant effect on improving the mixture resistance to rutting deformation. Rutting in flexible pavement can be divided into two types, consolidation rutting which happens with excessive consolidation of the pavement along the wheel path caused by decreased air voids in the asphalt concrete layer as shown in Figure 13, or the permanent deformation of the base or subgrade. Instability rutting is due to the asphalt mixture properties and is an occurrence in the range of the top 2 inches of the asphalt concrete layer as shown in Figure 14 [101].

## 9. Marshall Stability and Rubberised Asphalt

In relation to the plastic behaviour of materials, the stability of an asphaltic paving mixture is influenced by its internal friction, cohesion, and inertia. The friction component of stability in turn is governed by size, shape, gradation, and surface roughness of aggregate particles, intergranular contact, pressure due to compaction and loading, aggregate interlock caused by angularity, and viscosity of the binder. The cohesion depends on variables such as the rheology of the binder, number of contact points, density, and adhesion [102]. The results of Marshall Test by Samsuri [28] indicated that incorporation of rubber increases the Marshall stability and quotient. The increase varied with the form of rubber used and the method of incorporating the rubber into bitumen. The Marshall stability for mixes containing rubber powders was increased more than twofold and the Marshall quotient increased by nearly threefold compared to the normal unmodified bituminous mix. Mixes produced using bitumen preblended with fine rubber powders showed the greatest improvement rather than mixes produced by direct mixing of rubber with bitumen and aggregates. Thus, preblending of bitumen with rubber is a necessary step in order to produce an efficient rubberised bitumen binder probably due to adequate and efficient rubber dispersions in the bitumen phase. The optimum binder content was selected based on Marshall Mix design method as recommended by the Asphalt Institute [103] which uses five mix design criteria:

- lower Marshall stability,
- an acceptable average of Marshall flow,
- an acceptable average of air void,
- percent voids filled with asphalt (VFA),
- lower value of VMA.

### 9.1. Influence of Aggregate Gradation on Marshall Test

The mineral aggregate is bituminous concrete constituting about 95 percent of the mixture on a weight basic and about 85 percent on a volume basic. Characteristics of aggregate contributing to the properties of bitumen mixture would be gradation, particle surface texture, particles shape, cleanliness, and chemical composition [104]. Investigations showed that the effect of aggregate maximum size on the modified Marshall test results resulted in mixtures with aggregate maximum size of 19 mm leading to higher modified Marshall stability values and slightly decreased Marshall flow values than mixtures with aggregate maximum size of 38 mm. However, the disparity between the results for the two mixtures was minimal. Also, the modified Marshall flow did not present any specific trend for the two mixtures [105].

The aggregate maximum size had a marked effect on the amount of air voids and on the specific gravity of the specimens. Small percentages of air voids and higher values of air-cured specific gravity were obtained for mixture with 38 mm of aggregate maximum size compared to mixture with 19 mm of aggregate maximum size [105]. On the other hand, binder emulsion content showed a significant effect on the air voids and the specific gravity of the specimens. Increasing the binder emulsion content in the mixture filled the voids among aggregate particles and also allowed for more occurrence of compaction due to lubrication [105].

### 9.2. Influence of Compaction on Marshall Test

The stability values of the various mixes obtained using gyratory compaction were two to three times greater than the values obtained with Marshall Compaction. The flow values of the mixes obtained using gyratory compaction correlated with the stability values, where the maximum stability occurred the lowest with regard to the flow, while those obtained using the Marshall compaction were not consistent in this respect [106].

## 10. Asphalt Mixtures Testing 

Different tests and approaches have been used to evaluate asphalt concrete mixtures properties. Several material properties can be obtained from fundamental, mechanistic tests that can be used as input parameters for asphalt concrete performance models. The main aspects, which can be characterised using indirect tensile test, are resilient elastic properties, fatigue cracking, and the properties related to permanent deformation. The elastic stiffness of the asphalt mixtures can be measured using the indirect tensile test (IDT) [6, 107].

### 10.1. Indirect Tensile Strength Test

The indirect tensile strength of a sample is calculated from the maximum load to failure. According to Witczak et al. [108], the indirect tensile test (IDT) has been extensively used in the structural design of flexible pavements since the 1960s. Strategic Highway Research Program (SHRP) [109] recommended indirect tensile test for asphalt concrete mixture characterisation. The popularity of this test is mainly due to the fact that the test can be done using marshal sample or cores from filed. This test is easy, quick, and characterised as less variable. Guddati et al. [110] have also indicated that there is good potential in predicting fatigue cracking using indirect tensile strength results. A study was conducted to evaluate the performance of Polyethylene (PE) modified asphaltic mixtures based on physical and mechanical properties. Physical properties were evaluated in terms of penetration and softening point. The mechanical properties were evaluated based on the indirect tensile strength. The result presented that PE enhanced both physical and mechanical properties of modified binder and mixtures [9].

### 10.2. Resilient Modulus Test

The dynamic stiffness or “resilient modulus” is a measure of the load-spreading ability of the bituminous layers; it controls the levels of the traffic-induced tensile strains at the underside of the lowest bituminous bound layer which are responsible for fatigue cracking, as well as the stresses and strains induced in the subgrade that can lead to plastic deformations (O'Flaherty, 1988) [92]. The dynamic stiffness is computed by indirect tensile modulus test, which is a quick and nondestructive method. In general, the higher the stiffness, the better its resistance is to permanent deformation and rutting [28]. Eaton et al. [111] showed that the resilient modulus increased or the mix behaved in a stiffer manner (the mix becomes stronger) with a decrease in temperature; also, as the load time increased and the resilient modulus decreased or yielded more under a longer loading time. Indirect tensile resilient modulus test is widely used as a routine test to evaluate and to characterise pavement materials. Dallas and Kamyar [112] defined the resilient modulus as the ratio of the applied stress to the recoverable strain when a dynamic load is applied. In this test, a cyclic load of constant magnitude in the form of haversine wave is applied along the diametric axis of a cylindrical specimen for 0.1 seconds and has a rest period of 0.9 seconds, thus maintaining one cycle per second. Al-Abdul-Wahhab and Al-Amri [113] conducted a resilient modulus test on unmodified and modified asphalt concrete mixtures using Marshall specimen. A dynamic load of 68 kg was applied and stopped after a 100 load repetitions. The load application and the horizontal elastic deformation were used to compute the resilient modulus value. Two temperatures were used, 25°C and 40°C. The modified asphalt mixtures with 10% percent crumb rubber showed an improved modulus compared to the unmodified asphalt concrete mixtures.

### 10.3. Indirect Tensile Fatigue Test

**Figure 15:**
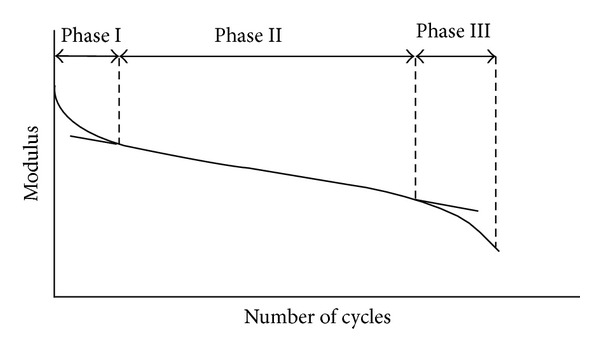
The three phases of fatigue test [115].

There are different test methods used throughout the world to measure fatigue resistance for asphalt concrete mixtures. Read [114] investigated the fatigue life of asphalt concrete mixtures using the indirect tension fatigue test. During the indirect tension fatigue, the horizontal deformation was recorded as a function of load cycle. The test specimen was subjected to different levels of stress, in order for a regression analysis on a range of values. This allows the development of the fatigue relationship between the number of cycles at failure (*N*_*f*_) and initial tensile strain (*ε*_*t*_) on a log-log relationship. Fatigue life (*N*_*f*_) of a specimen is number of cycles to failure for asphalt concrete mixtures. The fatigue life is defined as the number of load cycling application (cycles) resulting in either disintegration or a permanent vertical deformation. Fatigue test procedure is used to rank the bituminous mixture resistance to fatigue as well as a guide to evaluate the relative performance of asphalt aggregate mixture, to obtain data and input for estimating the structural behaviour in the road. During the fatigue test, modulus value decreased as indicated in Figure 15. Three phases were distinguished [115]:

- phase I: initially there is a rapid diminution of the modulus value,
- phase II: modulus variation is approximately linear,
- phase III: rapid decrease of the modulus value.

Damage is defined as any loss of strength that takes place in a specimen during a test.

A research study [18] investigated the fatigue behaviour of the different mixes using controlled-strain third-point flexural beam tests. Controlled-strain flexural fatigue testing indicated that the incorporation of CRMs in mixes can enhance their fatigue resistance. The magnitude of improvement appears to depend on the degree and type of rubber modification. Multilayer elastic analysis combined with fatigue test results for typical Alaskan conditions also indicated the enhanced fatigue behaviour of CRM mixes. However, condition surveys at both conventional and CRM sections revealed no longitudinal or alligator type of cracking, suggesting similar field fatigue performance for both materials.

## 11. Conclusion 

Today, a serious problem that leads to environment pollution is the abundance and increase of waste tyre disposal. Large amounts of rubber are used as tyres for cars and trucks, and so forth. Although rubber as a polymer is a thermosetting material cross-linked to processing and moulding, it cannot be softened or remoulded by reheating unlike other types of thermoplastics polymer which can be softened and reshaped when heated. Due to an increase in service traffic density, axle loading and low maintenance services road structures have deteriorated and are therefore subjected to failure more rapidly. To minimize the damage of pavement such as resistance to rutting and fatigue cracking, asphalt mixture modification is required. Virgin polymer offers the possibility of producing mixtures that can resist both rutting and cracking. Thus, using recycled polymer such as crumb rubber is a good alternative and inexpensive. Also, it is considered as sustainable technology, that is, “*greening asphalt*” which would transform unwanted residue into a new bituminous mixture highly resistant to failure. Thus, utilising crumb rubber obtained from scrap automobile tyre is not only beneficial in terms of cost reduction but also has less ecological impact in keeping the environment clean and to achieve better balance of natural resources.
